# Lymphocyte to C-reactive protein ratio is associated with in-hospital cardiac death in elderly patients with non-ST-segment elevation myocardial infarction

**DOI:** 10.3389/fcvm.2024.1431137

**Published:** 2024-08-13

**Authors:** Jun Luo, Han Shao, Yu Song, Yali Chao

**Affiliations:** ^1^ Department of Critical Care Medicine, The Affiliated Hospital of Xuzhou Medical University, Xuzhou, China; ^2^ Department of Anesthesiology, The Affiliated Hospital of Xuzhou Medical University, Xuzhou, China

**Keywords:** lymphocyte to C-reactive protein ratio, inflammation response, non-ST-segment elevation myocardial infarction, in-hospital cardiac death, elderly patients

## Abstract

**Background:**

Although percutaneous coronary intervention (PCI) is recommended by guidelines, data from the real world suggest that elderly non-ST-segment elevation myocardial infarction (NSTEMI) patients have a low rate of PCI and a high death rate. Lymphocyte to C-reactive protein ratio (LCR), a novel inflammatory marker, has been shown to be associated with prognosis in a variety of diseases. However, the relationship between LCR and in-hospital cardiac death in elderly NSTEMI patients is unclear. The aim of this study was to investigate the effect of LCR on in-hospital cardiac death in elderly NSTEMI patients without PCI therapy.

**Methods:**

This was a single-center retrospective observational study, consecutively enrolled elderly (≥75 years) patients diagnosed with NSTEMI and without PCI from February 2019 to February 2024. LCR was defined as lymphocyte count to C-reactive protein ratio. The endpoint of observation was in-hospital cardiac death. The predictive efficacy of the old and new models was evaluated by the net reclassification index (NRI) and the integrated discriminant improvement index (IDI).

**Results:**

A total of 506 patients were enrolled in this study, and in-hospital cardiac death occurred in 54 patients (10.7%). Univariate logistic regression analysis showed that left ventricular ejection fraction, LCR, Killip ≥2, and N-terminal B-type natriuretic peptide proteins (NT-proBNP) were associated with the occurrence of in-hospital cardiac death. After adjusting for potential confounders, the results showed that NT-proBNP (OR = 1.695, 95% CI: 1.238–2.322) and LCR (OR = 0.262, 95% CI: 0.072–0.959) were independent risk factors for in-hospital cardiac death. After the addition of LCR to NT-proBNP, the predictive ability of the new model for in-hospital cardiac death was significantly improved (NRI = 0.278, *P* = 0.030; IDI = 0.017, *P* < 0.001).

**Conclusion:**

Lower LCR is an independent risk factor for in-hospital cardiac death in elderly NSTEMI patients without PCI, and integrating LCR improves the prediction of in-hospital cardiac death occurrence.

## Introduction

Over the past decades, cardiovascular diseases (CVD) has remained the leading cause of death worldwide, placing enormous pressure and economic burden on healthcare systems (1). Currently, the incidence of CVD is on the rise due to the improvement of living standards and the aging of the population. Despite guidelines recommending early invasive strategies for high-risk non-ST-segment elevation myocardial infarction (NSTEMI) patients, these procedures are less common in the elderly due to varied adherence to the guidelines (2). Real-world data indicates that only one quarter of elderly patients with acute NSTEMI receive percutaneous coronary intervention (PCI), and most of these are elective procedures (3). Previous studies showed that patients aged ≥75 years account for up to 24.7% of patients with NSTEMI and have a in-hospital death rate of 11.5% (4, 5). Therefore, as a high-risk group of concern, the use of simple and reliable biomarkers to predict adverse events in elderly NSTEMI patients will help optimize risk stratification, and early intervention and improve prognosis.

Inflammatory responses could promote the development of a variety of diseases and were associated with higher mortality, including in patients with CVD (6). In addition to lipid factors, the inflammatory response played an important role in thrombosis induced by atherosclerotic plaques (7). Recently, the lymphocyte-to-C-reactive protein ratio (LCR) has emerged as a new and valuable biomarker of inflammation as a useful tool for predicting the outcome of various diseases and guiding therapeutic decisions, including cancer, myocardial injury, and COVID-19 (8–11). LCR was previously shown to be a novel and valuable parameter for assessing postoperative infection-related mortality in cardiac surgery patients (12). A recent study showed that LCR was associated with new-onset AF in STEMI patients treated with pPCI (13). In addition, some other studies indicated that a lower LCR was an independent protective factor against CAD development and severity, as well as being positively associated with MI prevalence (14, 15). However, the relationship between LCR and in-hospital cardiac death in patients with NSTEMI remains unclear.

The aim of this study was to investigate the predictive value of LCR for the risk of in-hospital cardiac death in elderly NSTEMI patients.

## Methods

### Study population

**Figure 1:**
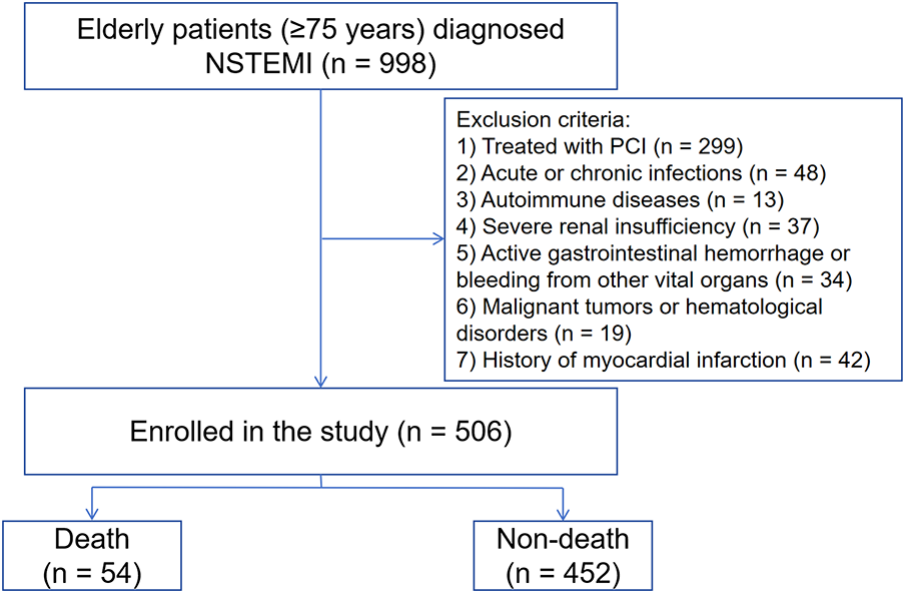
Study flowchart. STEMI, ST-segment elevation myocardial infarction. PCI, percutaneous coronary intervention.

This was a single-center retrospective clinical observational study. We consecutively selected patients who were diagnosed with NSTEMI (16) at the Affiliated Hospital of Xuzhou Medical University from February 2019 to February 2024. Inclusion criteria: age ≥75 years. Exclusion criteria: (1) treated with PCI; (2) patients with acute or chronic infections; (3) patients with autoimmune diseases; (4) severe hepatic and renal insufficiency (estimated glomerular filtration rate <30 ml/min/1.73 m^2^); (5) active gastrointestinal hemorrhage or bleeding from other vital organs; (6) patients with malignant tumors or hematological disorders; and (7) patients with a previous history of myocardial infarction (MI). The Institutional Review Board (IRB) of the Affiliated Hospital of Xuzhou Medical University approved this study protocol. The requirement for signed written consent was waived owing to the low risk to the patient in accordance with the relevant IRB regulatory guidelines. Finally, 506 patients were enrolled in the study. Patient enrollment in this study is shown in Figure 1.

### Clinical data collection

Clinical information was collected from all patients, including general clinical characteristics and past medical history. Lymphocyte and C-reactive protein (CRP) indices were recorded for patients on the day of admission. LCR was defined as lymphocyte count to C-reactive protein ratio. Lipid-related indices were the results of the first fasting laboratory test after admission. The peak values of high-sensitivity troponin T (hs-TnT) and N-terminal B-type natriuretic peptide proteins (NT-proBNP) during hospitalization were collected. Medication use during hospitalization was recorded for all patients. The primary endpoint was in-hospital cardiac death (defined as any death of cardiac origin). Patients was divided into two groups based on the mortality outcome.

### Statistical analysis

All data were statistically analyzed using SPSS (version 26.0, Chicago, USA) and R 4.3.1. The normality of data was determined using the Kolmogorov-Smirnov test. Continuous variables that conformed to a normal distribution were expressed as mean ± standard deviation and analyzed using the independent samples *t*-test. Continuous variables that were not normally distributed were described as median [interquartile range (IQR)] and were analyzed using the Mann-Whitney *U*-test. Categorical variables were expressed as counts and percentages and analyzed statistically using the χ^2^ test. Potential risk factors identified by univariate analysis and clinically significant variables were included in multivariate analysis using the stepwise forward method to determine independent risk factors associated with in-hospital cardiac death. The predictive efficacy of all models was evaluated by receiver operating characteristic (ROC) and the net reclassification index (NRI) and the integrated discriminant improvement index (IDI). *P* < 0.05 was considered a statistically significant difference.

## Results

### Patient characteristics

The incidence of in-hospital cardiac death was 54/506 (10.7%). The patients were classified into events and non-events groups. A comparison of the clinical data between the two groups is shown in Table 1. Compared with the without events group, the with events group had a higher proportion of Killip ≥2, hs-TnT, NT-proBNP, and lower left ventricular ejection fraction (LVEF) and LCR [0.041 (0.016, 0.230) vs. 0.156 (0.031, 0.365), *P* = 0.001] (all *P* < 0.05) (Table 1).

**Table 1:** Patient characteristics between events and non-event groups.

	With events (*n* = 54)	Without events (*n* = 452)	*P*
Age (years)	80.19 ± 4.17	79.53 ± 3.68	0.226
Male, *n* (%)	34 (63.0)	302 (66.8)	0.571
BMI (kg/m^2^)	22.17 ± 4.62	21.79 ± 3.35	0.447
Heart rate (bpm)	81.56 ± 14.41	79.21 ± 14.09	0.250
SBP (mmHg)	116.59 ± 23.41	116.97 ± 20.48	0.899
DBP (mmHg)	71.46 ± 15.11	73.00 ± 13.97	0.450
Hypertension, *n* (%)	31 (57.4)	211 (46.7)	0.136
Diabetes, *n* (%)	18 (33.3)	135 (29.9)	0.600
Stroke, *n* (%)	9 (16.7)	73 (16.2)	0.922
Current smoker, *n* (%)	21 (38.9)	187 (41.4)	0.726
LVEF (%)	41.41 ± 6.58	43.90 ± 6.55	0.008
Killip ≥2, *n* (%)	21 (38.9)	107 (23.7)	0.015
Laboratory indicators			
Triglycerides (mmol/L)	4.35 ± 0.99	4.42 ± 1.01	0.617
Total cholesterol (mmol/L)	1.71 ± 1.47	1.47 ± 0.92	0.087
LDL-C (mmol/L)	2.55 ± 0.67	2.76 ± 0.87	0.082
HDL-C (mmol/L)	1.08 ± 0.26	1.04 ± 0.23	0.227
LCR	0.041 (0.016, 0.230)	0.156 (0.031, 0.365)	0.001
NT-proBNP (pg/ml)	4,466.0 (2,361.5, 8,778.6)	2,323.1 (1,416.6, 4,346.0)	<0.001
hsTnT (ng/L)	4,863.0 (1,770.3, 10,000.0)	3,755.0 (1,609.3, 6,388.8)	0.030
Medicines			
P2Y12 inhibitor, *n* (%)	50 (92.6)	433 (95.8)	0.285
Aspirin, *n* (%)	48 (88.9)	416 (92.0)	0.428
Statin, *n* (%)	50 (92.6)	425 (94.0)	0.678
SGLT2 inhibitors, *n* (%)	14 (25.9)	96 (21.2)	0.430
ACEI/ARB, *n* (%)	24 (44.4)	228 (50.4)	0.405
Beta-blocker, *n* (%)	41 (75.9)	342 (75.7)	0.995
Spironolactone, *n* (%)	12 (22.2)	61 (13.5)	0.085

BMI, body mass index; SBP, systolic blood pressure; DBP, diastolic blood pressure; LVEF, left ventricular ejection fraction; LDL-C, low-density lipoprotein-cholesterol; HDL-C, high-density lipoprotein-cholesterol; LCR, lymphocyte count to C-reactive protein ratio; hsTnT, high-sensitivity troponin T; NT-proBNP, N-terminal B-type natriuretic peptide proteins; ACEI, angiotensin converting enzyme inhibitors; ARB, angiotensin receptor blocker.

### Relationship between LCR and in-hospital cardiac death

Univariate logistic regression analysis showed that LVEF, LCR, Killip ≥2, and NT-proBNP were associated with the occurrence of in-hospital cardiac death (*P* < 0.05). The above variables were included in a multivariate logistic regression, and the results showed that NT-proBNP (OR = 1.695, 95% CI: 1.238–2.322) and LCR (OR = 0.262, 95% CI: 0.072–0.959) were independent factors influencing the occurrence of in-hospital cardiac death (Table 2).

**Table 2:** Logistic regression analysis on in-hospital cardiac death.

	Univariate analysis			Multivariate analysis		
	OR	95% CI	*P*	OR	95% CI	*P*
Age (years)	1.045	0.973–1.122	0.227			
Male, *n* (%)	1.184	0.659–2.128	0.572			
BMI (kg/m^2^)	1.031	0.953–1.116	0.446			
Heart rate (bpm)	1.012	0.992–1.032	0.250			
SBP (mmHg)	0.999	0.986–1.013	0.899			
DBP (mmHg)	0.992	0.972–1.012	0.449			
Hypertension, *n* (%)	1.539	0.870–2.723	0.138			
Diabetes, *n* (%)	1.174	0.644–2.141	0.600			
Stroke, *n* (%)	1.038	0.486–2.216	0.922			
Current smoker, *n* (%)	0.902	0.506–1.608	0.726			
LVEF (%)	0.945	0.906–0.986	0.009			
Killip ≥2, *n* (%)	2.052	1.139–3.696	0.017			
Triglycerides (mmol/L)	0.930	0.700–1.236	0.617			
Total cholesterol (mmol/L)	1.215	0.966–1.529	0.096			
LDL-C (mmol/L)	0.733	0.516–1.041	0.083			
HDL-C (mmol/L)	1.993	0.650–6.114	0.228			
LCR	0.226	0.062–0.832	0.025	0.262	0.072–0.959	0.043
NT-proBNP (pg/ml)	1.902	1.418–2.551	<0.001	1.695	1.238–2.322	<0.001
hsTnT (ng/L)	1.331	0.953–1.859	0.093			

BMI, body mass index; SBP, systolic blood pressure; DBP, diastolic blood pressure; LVEF, left ventricular ejection fraction; LDL-C, low-density lipoprotein-cholesterol; HDL-C, high-density lipoprotein-cholesterol; LCR, lymphocyte count to C-reactive protein ratio; hsTnT, high-sensitivity troponin T; NT-proBNP, N-terminal B-type natriuretic peptide proteins.

### Receiver operating characteristic analysis on in-hospital cardiac death

**Figure 2:**
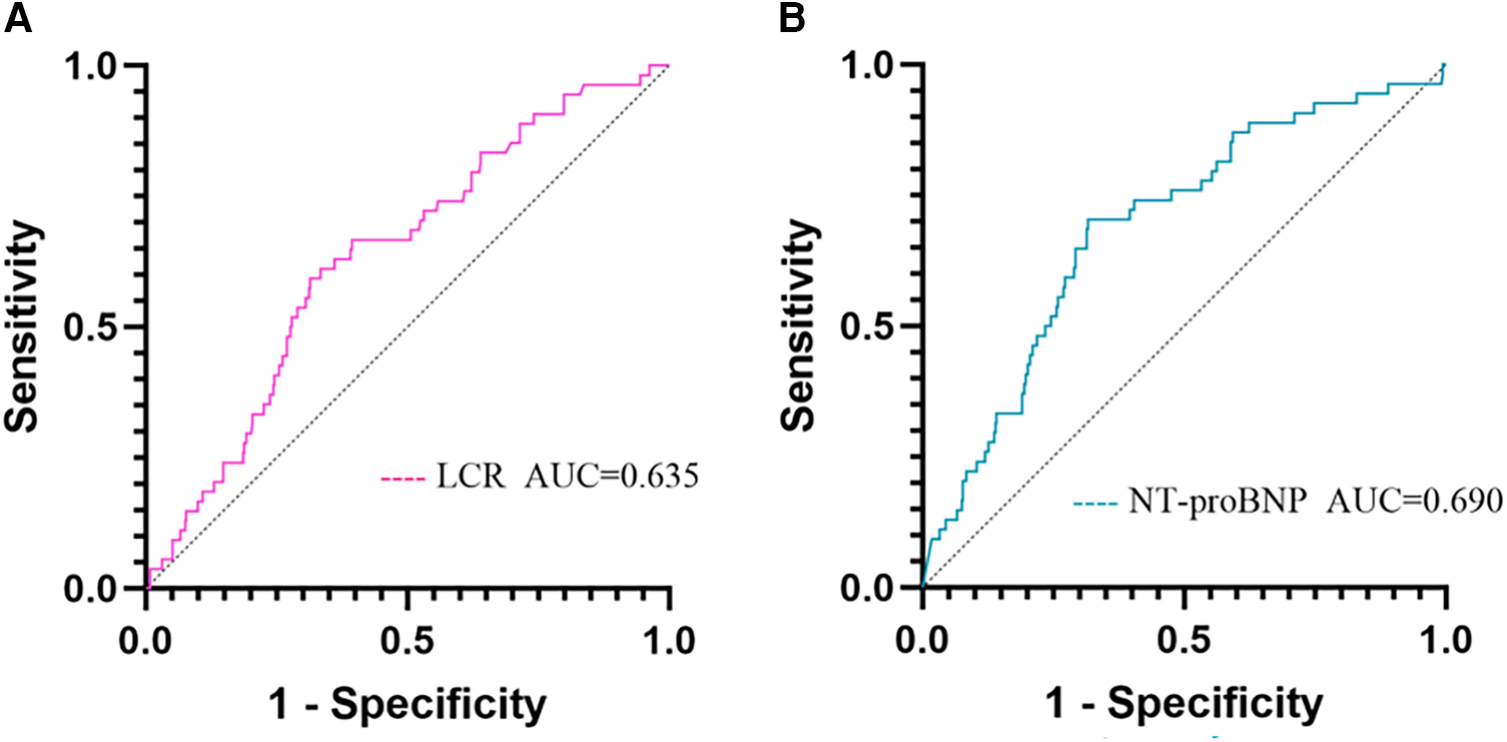
ROC analysis for predicting in-hospital cardiac death. (**A**) ROC analysis of LCR predicting in-hospital cardiac death; (**B**) ROC analysis of NT-proBNP predicting in-hospital cardiac death. LCR, lymphocyte count to C-reactive protein ratio; NT-proBNP, N-terminal B-type natriuretic peptide proteins.

As shown by the ROC analysis, the areas under the curves (AUC) of NT-proBNP and LCR for predicting the occurrence of in-hospital cardiac death were 0.690 and 0.635, respectively, corresponding to a *P*-value of <0.05, which was statistically different. In particular, the cutoff value for LCR to predict in-hospital cardiac death was 0.057, corresponding to a sensitivity of 59.3% and a specificity of 68.6% (Table 3, Figure 2).

**Table 3:** ROC analysis for predicting in-hospital cardiac death.

Variables	AUC	Cut-off	95% CI	Sensitivity	Specificity	*P*
LCR	0.635	0.057	0.592–0.677	0.593	0.686	0.032
NT-proBNP	0.690	3,484.8	0.648–0.731	0.704	0.684	<0.001

LCR, lymphocyte count to C-reactive protein ratio; NT-proBNP, N-terminal B-type natriuretic peptide proteins.

### Incremental value of LCR for predicting in-hospital cardiac death

**Figure 3:**
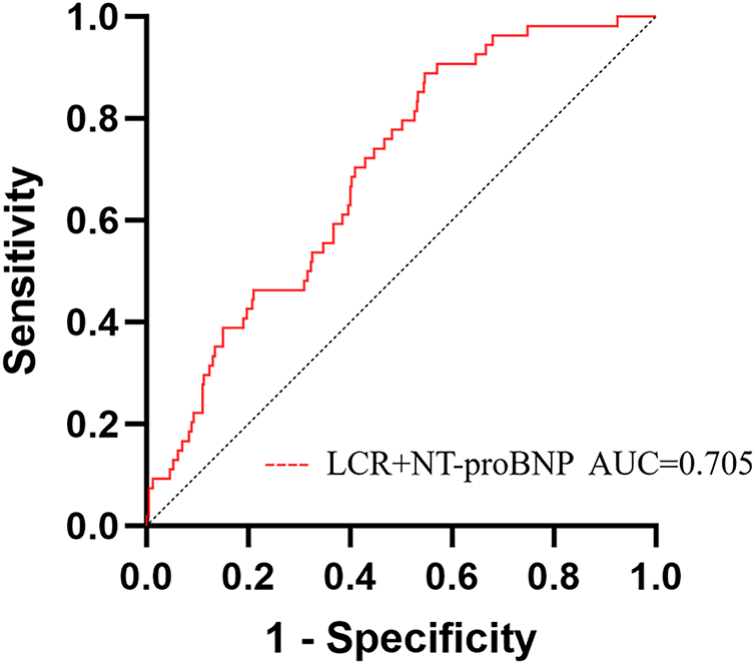
ROC analysis of a new model (NT-proBNP + LCR) for predicting in-hospital cardiac death in elderly NSTEMI patients. LCR, lymphocyte count to C-reactive protein ratio; NT-proBNP, N-terminal B-type natriuretic peptide proteins.

A new model was established by combining LCR with NT-proBNP, and ROC curves were plotted. The results showed that the AUC of the new model was 0.705 (95% CI: 0.630–0.861, *P* < 0.001), corresponding to a sensitivity of 88.9% and a specificity of 65.4%. The NRI and IDI indices of the traditional model and the new model were calculated and compared, with NRI = 0.278 (95% CI: 0.027–0.529, *P* = 0.030), IDI = 0.017 (95% CI: 0.009–0.025, *P* < 0.001). The results indicated that the ability of the new model to predict in-hospital cardiac death was significantly improved (Table 4, Figure 3).

**Table 4:** Incremental value of LCR for predicting in-hospital cardiac death.

	IDI (95% CI)	*P*	NRI (95% CI)	*P*
NT-proBNP	–	–	–	–
NT-proBNP + LCR	0.017 (0.009–0.025)	<0.001	0.278 (0.027–0.529)	0.030

LCR, lymphocyte count to C-reactive protein ratio; NT-proBNP, N-terminal B-type natriuretic peptide proteins; NRI net reclassification index; IDI, integrated discriminant improvement index.

## Discussion

To the best of our knowledge, this study was the first to investigate the predictive value of LCR for the risk of in-hospital cardiac death in elderly NSTEMI patients. The main findings of this study are as follows. First, LCR was significantly lower in the with events group compared with the without events group; Second, a lower LCR was an independent risk factor for in-hospital cardiac death in elderly NSTEMI patients; Third, LCR had a diagnostic value for in-hospital cardiac death in elderly NSTEMI patients; Fourth, the integration of LCR could significantly improve the risk model about in-hospital cardiac death in elderly NSTEMI patients.

Recent data indicate that CVD remain the leading cause of death in the population, with the mortality rate for patients with acute myocardial infarction still being significantly high (17). Studies from the real world showed that the low rate of PCI therapy in elderly NSTEMI patients was accompanied by high mortality (3). Similarly, in this study, the incidence of in-hospital cardiac death in elderly NSTEMI patients without PCI therapy was 10.7%. Therefore, as a unique population, there is still a need to explore more risk factors in elderly NSTEMI patients to identify high-risk patients early and optimize risk stratification.

Inflammation is known to play an important role in cardiovascular disease. Although inflammatory markers such as IL-6, TNF-a, and MMP-9 have been shown to be sensitive and reliable predictors of poor prognosis (18), these tests are usually not routinely available, limiting their use in clinical practice. The complete blood count is a simple and commonly used measurement that can help predict inflammation, and combinations of several hematologic markers, such as the neutrophil-to-lymphocyte ratio (NLR), have been developed as prognostic markers in patients with AMI (19–21). In recent years, LCR has been widely demonstrated to be significantly associated with prognosis as a new indicator of inflammation in cancer patients (22). In the cardiovascular field, LCR is independently and negatively correlated with subclinical myocardial injury (8). In addition, Gao et al. (13) found that preoperative LCR was an independent predictor of new-onset AF in patients with acute ST-segment elevation myocardial infarction after PCI. The results of this study are consistent with the published literature that low LCR was an independent risk factor for the occurrence of in-hospital cardiac death in elderly NSTEMI patients. This may be related to the following factors. CRP is a widely accepted universal inflammatory marker, and CRP levels were elevated in response to cellular damage or tissue injury (23). In clinical applications, CRP appears to be the most promising biomarker of inflammation, and many population-based studies have shown that initial CRP levels could be used to predict cardiovascular events (24). Previous studies showed that CRP was involved in the pathogenesis of AMI by influencing complement activation and causing vascular endothelial dysfunction and that elevated CRP levels in patients with AMI were associated with a poor prognosis (25, 26). Lymphocytes played an important role in the immune response, and lymphopenia often indicates a poor prognosis (27). In MI, the body is in a state of stress, and elevated levels of catecholamines and cortisol lead to an increase in lymphocyte apoptosis, decreasing in lymphocyte count (28). Previous studies confirmed that decreased peripheral blood lymphocyte counts in patients with acute chest pain were associated with the progression of atherosclerosis, impaired coronary microcirculation, and the occurrence of major cardiac events (29, 30). The LCR is determined by two indicators: lymphocyte counts and CRP levels. Based on this information, elevated CRP and decreased lymphocyte counts, leading to a decreased LCR, indicate an increased systemic inflammatory response, which may lead to a poor prognosis in elderly NSTEMI patients.

In future studies, LCR may receive increasing attention as a novel and easily accessible indicator. As found in this study, LCR had a good predictive value for in-hospital cardiac death in elderly NSTEMI patients, and after integrating LCR, the predictive ability of the new model (NT-proBNP + LCR) for in-hospital cardiac death in elderly NSTEMI patients was significantly improved. This may help to optimize the risk stratification of this group of patients, leading to early intervention and improved clinical prognosis.

This study has some limitations. First, this was a single-center retrospective study with a small sample size, and there may be some unidentified confounders in the obtained data, which may lead to bias. Second, although the results of this study suggest that the prognosis of elderly NSTEMI patients in the low LCR group was poorer, the specific mechanism of action may need to be further explored by future basic research. Third, this study is limited to examining in-hospital mortality and does not consider the impact on long-term outcomes post-discharge; future research is required to ascertain whether LCR has an influence on long-term outcomes among these patients' population. Finally, this study only investigated the relationship between LCR and in-hospital cardiac death in elderly NSTEMI patients; therefore, the findings of this study may not be directly applicable to other populations.

## Conclusion

Lower LCR is an independent risk factor for in-hospital cardiac death in elderly NSTEMI patients, and the integration of LCR could significantly improve the risk model for in-hospital cardiac death in this patient group. This implies that LCR may be useful for assessing inflammation and predicting cardiovascular disease risk stratification in elderly NSTEMI patients.

## Data Availability

The raw data supporting the conclusions of this article will be made available by the authors, without undue reservation.
